# Enhancing mRNA translation efficiency with discriminative and generative artificial intelligence by optimizing 5′ UTR sequences

**DOI:** 10.1016/j.isci.2025.113544

**Published:** 2025-09-10

**Authors:** Yu Liu, Chunmei Cui, Limei Liu, Qinghua Cui

**Affiliations:** 1 Department of Biomedical Informatics, State Key Laboratory of Vascular Homeostasis and Remodeling, School of Basic Medical Sciences, Peking University, 38 Xueyuan Road, Beijing 100191, China; 2 Department of Physiology and Pathophysiology, State Key Laboratory of Vascular Homeostasis and Remodeling, School of Basic Medical Sciences, Peking University, 38 Xueyuan Road, Beijing 100191, China; 3 School of Sports Medicine, Wuhan Institute of Physical Education, No. 461 Luoyu Road Wuchang District, Wuhan 430079, Hubei Province, China

**Keywords:** Biochemistry, artificial intelligence

## Abstract

The mRNA-based therapeutics, notably mRNA vaccines, represent a new era of powerful tools to combat various diseases. However, the relatively low translation efficiency of exogenous mRNA often limits its wide application. Here, we propose a computational framework called UTailoR (UTR tailor), which significantly improves the challenge by optimizing 5′ UTR sequences based on a two-step artificial intelligence strategy. We first develop a deep-learning-based discriminative model for predicting mRNA translation efficiency with 5′ UTR sequences and then present a generative model to generate optimized 5′ UTR sequences, which are designed to be highly close to the original sequences but predicted to result in high translation efficiency. The experimental results show that the UTailoR-optimized sequences outstrip the corresponding original sequences by ∼200%. This work provides an efficient and convenient method for mRNA 5′ UTR optimization, which can be easily accessed online.

## Highlights

• The discriminative model can accurately predict the translation efficiency of 5′ UTR
• The generative model can generate 5′ UTRs tailored to specific mRNA sequences
• UTailoR online service has been developed for easily optimizing 5′ UTRs

## Graphical abstract

**Figure:**
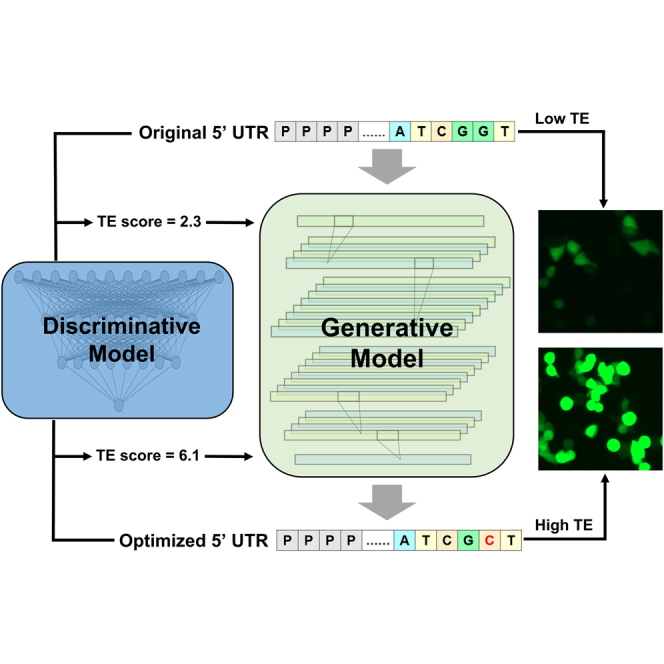

## Introduction

In recent years, mRNA-based therapeutics, notably mRNA vaccines, have been extensively utilized in the treatment of various diseases, including cancer, cardiovascular disease, and infectious diseases.^1^^,^^2^ Compared with conventional approaches, mRNA-based therapeutics offer enhanced safety profiles, accelerated design and production processes, as well as reduced costs.^3^^,^^4^ However, one major challenge limiting the widespread application of this therapeutics is that the exogenous mRNAs within the human body often show low translation efficiency (TE).^5^ Therefore, it is quite important to develop *in silico* approaches to optimize mRNA sequence to improve its translation efficiency without altering the corresponding protein sequence.

mRNA sequence consists of coding sequence (CDS) and untranslated region (UTR). Currently, the optimization of CDS has been extensively investigated, encompassing methods based on codon optimality theory^6^ as well as those based on deep learning.^7^ However, it is widely acknowledged that mRNA translation efficiency is influenced not only by the CDS but also by the UTR, especially the 5′ UTR sequence as it directly impacts ribosome recruitment and binding, serving as a primary determinant of translation efficiency.^8^^,^^9^^,^^10^ However, due to the limited understanding of the function of 5′ UTR, few studies on its optimization have been developed. Currently, there are two main strategies for optimizing 5′ UTR. One is based on prior knowledge, that is, utilizing known 5′ UTRs with high translation efficiency.^11^ The other is a genetic algorithm-based approach, which can iteratively evolve 5′ UTR sequences to obtain enhanced translation efficiency.^12^ However, both methods aim to obtain a small number of universally applicable sequences while disregarding gene-specific differences and sequence information. Consequently, they often fail to achieve optimal performance. In light of this limitation, it becomes necessary to develop an optimization method capable of designing distinct 5′ UTR sequences with high mRNA translation efficiency tailored for specific genes.

In recent years, deep learning methods have been extensively applied to biological and medical problems. Deep learning methods have made breakthrough progress in numerous transcription and translation-related issues, including transcription start site prediction,^13^ transcription factor prediction,^13^ and mRNA degradation prediction,^14^ and have now become powerful tools for solving various biological problems. The massively parallel reporter assay (MPRA) is a method based on high-throughput sequencing, which can simultaneously measure the translation efficiency of hundreds of thousands of mRNA sequences encoding the same reporter gene but with different UTRs.^15^ MPRA enables the acquisition of a large dataset consisting of 5′ UTR sequences and their corresponding translation efficiency values,^16^ facilitating the application of deep learning methods to the problem of translation efficiency. One previous study utilized this dataset to develop deep learning models for predicting translation efficiency based on the 5′ UTR sequence, which demonstrated excellent performance^17^; however, it only provided a small number of universally applicable sequences instead of gene-specific distinct 5′ UTR sequences, which limited the translation efficiency of most mRNAs. In this study, we propose a two-step computational framework called UTailoR to solve the aforementioned problem. We draw inspiration from generative adversarial network principles^18^ to train a discriminative model that predicts translation efficiency, which thereby guides us to develop a generative model to generate 5′ UTR sequences that are highly close to the original ones but with enhanced translation efficiency. Compared with conventional approaches, UTailoR employs deep learning strategies to explore sequence features associated with high translation efficiency while generating tailored 5′ UTR sequences for specific genes, which effectively preserves the inherent characteristics of the original sequence and exhibits enhanced versatility.^19^ Ultimately, we develop an online tool for optimizing mRNA 5′ UTR sequences, which can be accessed freely at http://www.cuilab.cn/utailor.

## Results

### The discriminative model accurately predicts translation efficiency

**Figure 1:**
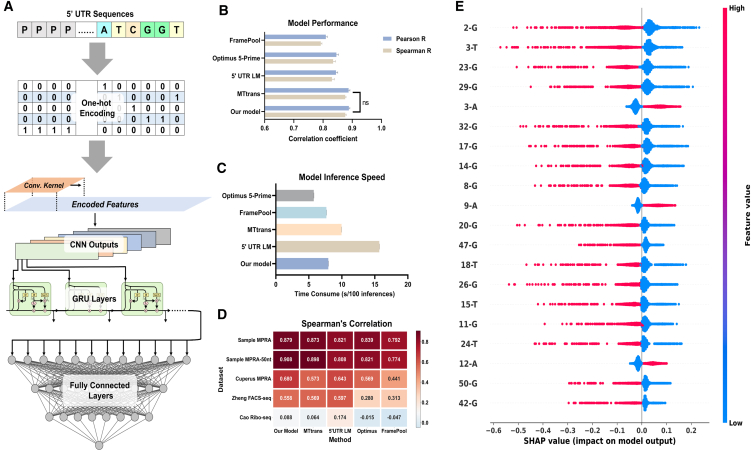
Structure and performance of the discriminative model in the UTailoR framework (A) Diagram illustrating the architecture of the discriminative model. One-hot encoded features undergo one-dimensional convolution and go through GRU units successively, where the number of GRU units corresponds to the length of the convolutional features. The output from the final GRU unit serves as input for fully connected layers. (B) Performance comparison of various models on a variable-length test set derived from HEK293T cells. Error bars represent standard deviation calculated from 10 experimental results. (C) Comparative analysis of computational speed across different models. A total of 100 randomly selected sequence features are individually inputted into each model, and the aggregate time taken by each model is recorded over 10 experiments. Data are represented as mean ± SD. (D) Heatmap illustrating the Spearman’s correlation coefficient between predicted and measured translation efficiency for each method across 5 datasets. All models were trained using Sample MPRA dataset. (E) The top 20 features ranked by absolute mean value of SHAP value, and the relationship between the feature value and SHAP value for each feature.

Currently, there have been some deep-learning-based methods for predicting translation efficiency.^16^^,^^17^^,^^20^^,^^21^ However, these methods address multiple downstream tasks, resulting in large parameter sizes and computational challenges during model training. Here, we developed a lightweight model specifically designed for predicting mean ribosome loading (MRL). This model solely utilizes the encoded features of the 5′ UTR sequence as an input, which undergo three layers of residual-connected convolutional layers, one Gate Recurrent Unit (GRU) layer, and three residual-connected fully connected layers to output predicted MRL scores (Figure 1A), which serve as an indicator for characterizing the translation efficiency of mRNA sequences.^22^^,^^23^

After optimizing hyperparameters and weights (see STAR Methods), our model achieved performance comparable to the current state-of-the-art methods (Table 1), with the Spearman’s correlation coefficient between predicted and actual values reaching up to 0.878 (Figure 1B; Table S3). Meanwhile, the running time of our model is approximately 50% shorter than that of the 5′ UTR LM method based on a large language model (Figure 1C; Table S3). Subsequently, we assessed our model using different datasets (Table 2). The results demonstrated its robust performance across various MPRA datasets (Figure 1D; Figure S2). Notably, despite being trained on enhanced green fluorescent protein (EGFP) data from the HEK293T cell line, our model exhibited strong performance on the yeast MPRA dataset, indicating that the impact of 5′ UTR sequences on translation efficiency can be generalized across genes and even species.^24^ Nevertheless, no significant correlation was observed between the predicted values and the true values for all methods on the Ribo-seq dataset (Figure 1D). We postulate that this discrepancy may stem from the absence of consistent control in CDS regions within the Ribo-seq data, thereby resulting in translation efficiency being influenced by both CDS and UTR.^25^^,^^26^^,^^27^

**Table 1:** Summary of the baseline discriminative methods

Name	Year	Method	Author	PMID
Optimus 5-Prime	2019	CNN	P. J. Sample	Sample et al.^16^
FramePool	2021	CNN+Pooling	A. Karollus	Karollus et al.^20^
MTtrans	2023	CNN+GRU	W. Zheng	Zheng et al.^17^
5′ UTR LM	2024	Transformer	Y. Chu	Chu et al.^21^

**Table 2:** Summary of the datasets used in this study

Author	Year	Biological material	Method	UTR length	TE format	Size
Sample et al.^16^	2019	HEK293T cell	MPRA	50 nt	mean ribosome loading	145,251
Sample et al.^16^	2019	HEK293T cell	MPRA	25–100 nt	mean ribosome loading	87,000
Cuperus et al.^28^	2017	*S. cerevisiae*	MPRA	50 nt	Log2 growth rate	500,000
Cao et al.^12^	2021	HEK293T/PC3/muscle	Ribo-seq	120 nt	RPKM ratio	6,721
Zheng et al.^17^	2023	HEK293T/hES cell	FACS-seq	100 nt	fluorescence intensity percentile	3,179

In addition, we used Shapley additive explanations (SHAP) to evaluate the importance of each input feature and paid attention to how the most important features affect the prediction results. The results revealed that most of the top-ranked features were T and G nucleotides upstream of the CDS region (we define the first position upstream of the start codon as “1” and the second position as “2,” and so on), exerting a negative influence on translation efficiency (Figure 1E; Figure S3). This aligns with existing knowledge, as ATG in the UTR forms an upstream open reading frame that hinders recognition of the main open reading frame by the ribosome, thereby reducing translation efficiency.^9^

### The generative model generates optimized 5′ UTR sequences with higher MRL scores

**Figure 2:**
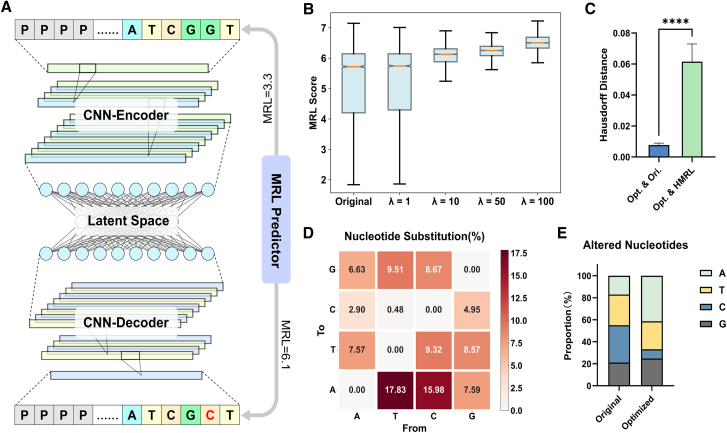
Structure and performance of the generative model in the UTailoR framework (A) Schematic diagram of the structure of the generative model, where “MRL Predictor” refers to the discriminative model in Figure 1A. MRL Predictor predicts the MRL score of the input sequence and the output sequence and then calculates the RL loss for updating the weight of the generative model. (B) Comparison of the MRL scores of the generated sequences and the original sequences on the variable-length 5′ UTR test set, where λ represents the weight of the RL loss in the loss function. The boxplot shows the first quartile and the third quartile of each group of data, and the whiskers represent the upper and lower bounds of the data. (C) The Hausdorff distance from optimized sequences to original sequences, and to known high-translation-efficiency sequences in the t-SNE space. 5 random samples are taken, with a sample size of 100 for each kind of sequence. Data are represented as mean ± SD, ∗∗∗∗*p* < 0.0001. (D) Heatmap illustrating the categories of nucleotide substitutions before and after sequence optimization, with the numbers in the boxes denoting the proportion of each substitution out of all substitution nucleotides. (E) Cumulative bar chart showing the proportion of altered nucleotide in original sequences and optimized sequences.

As previously mentioned, the current general approach to optimizing 5′ UTR sequences is to search for “universally applicable” sequences without considering the information of the original UTR sequence for specific genes.^29^^,^^30^ Therefore, these methods limit the exploration of higher translation efficiency sequences to some extent. So here we attempted to develop a method that could generate optimized sequences with enhanced translation efficiency while maintaining similarity to the original reference sequence as much as possible. Ultimately, we developed a special autoencoder-based generative model, which we call the “Generative Autoencoder” (Figure 2A). The loss function of this model comprises two components: the reconstruction loss ensures that the generated sequence closely resembles the original sequence, while the RL (representing “ribosome loading”) loss guides the model in producing sequences with high MRL scores (see STAR Methods).

After appropriately adjusting the weights of the two parts of the loss function and training the model, the model achieved the expected results (Tables S4 and S5). As the weight of the RL loss was increased, the model tended to generate sequences with higher MRL scores (Figure 2B; Figure S4; Table S6). Concurrently, the results of the t-distributed stochastic neighbor embedding (t-SNE) dimensionality reduction analysis showed that the generated sequences were more similar to the original sequences than the known high MRL sequences (*p* < 0.0001, Student’s t test, Figure 2C; Figure S5). To sum up, the model has indeed learned rules affecting translation efficiency and can generate entirely new high-efficiency sequences based on these rules, rather than attempting to find a sequence in known high-efficiency sequences most similar to the query sequence.

Next, we evaluated the differences between the generated sequences and the original sequences. For most sequences, the generative model only changed 4–10 nucleotides, with the most common conversions being T-to-A and C-to-A (Figure 2D). Ultimately, the adenine content of the optimized sequence increased from the original 17.1% to 41.4% across all mutated sites (Figure 2E; Table S7), which was consistent with the bias of the discriminative model for adenine in the feature importance analysis in Figure 1E.

### The generative model-optimized 5′ UTR sequences enhance translation efficiency

**Figure 3:**
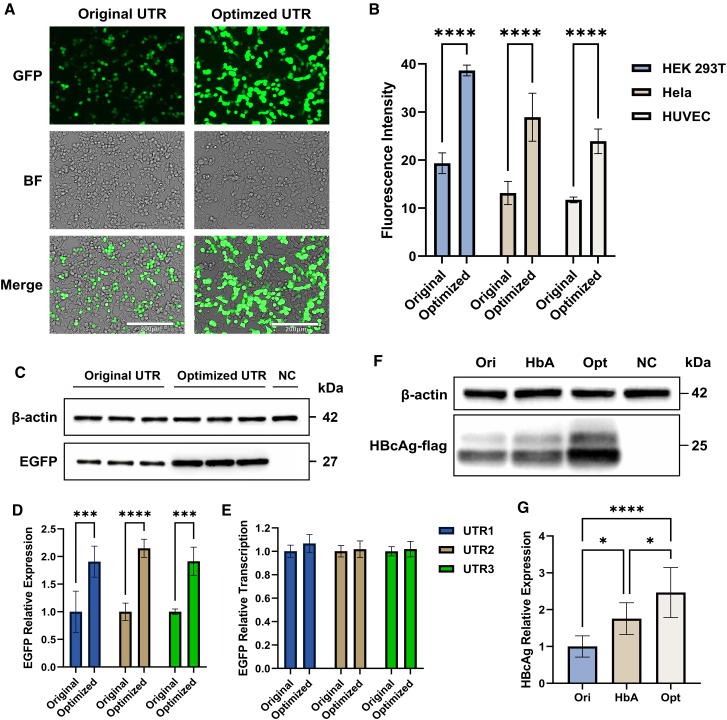
UTailoR-optimized 5′ UTR sequence enhances the translation efficiency of EGFP (A) Fluorescence microscopy images of HEK293T cell, taken 24 h after transfection, magnified 400×. BF, bright field. (B) Statistical analysis of the mean fluorescence intensity in fluorescence microscopy, with 5 fields of view taken from each sample and 3 samples (i.e., Ori1–3 and Opt1–3) per group. (C) Representative western blot image of Ori-cell and Opt-cell EGFP expression, from left to right: Ori1-3 and Opt1-3. NC represents transfection of the empty vector as a negative control. (D) Differences in EGFP expression between Ori-cell and Opt-cell, with 4 samples per group, and the error bars represent the standard deviation. (E) Differences in EGFP mRNA content between Ori-cell and Opt-cell, with 4 samples per group, and the error bars represent the standard deviation. (F) Representative western blot image of HBcAg expression. Ori, original HBcAg UTR; HbA, human alpha globin UTR; Opt, optimized UTR by UTailoR; NC, negative control. (G) Differences in HBcAg expression among the three groups of cells, with 4 samples per group, and the error bars represent the standard deviation. Data are represented as mean ± SD, ∗*p* < 0.05, ∗∗∗*p* < 0.001, and ∗∗∗∗*p* < 0.0001, Student’s t test (B, D, and E) or one-way ANOVA (G).

To validate the computational results generated by our generative model, we selected the three pairs of sequences with the highest predicted translation efficiency improvement for experimental verification (Table S1). We transfected these UTR-EFGP plasmid into HEK293T cells, HeLa cells, and HUVECs, respectively, and then evaluated the fluorescence intensity. The results revealed comparable trends across all tested sequences and cell lines (Figure 3A; Figures S6 and S7). The fluorescence intensity of cells transfected with the original sequence and optimized sequence (here named as “Ori-cell” and “Opt-cell,” respectively) reached its peak at 36–48 h after transfection (Figure S8), and the fluorescence intensity of Opt-cell was significantly higher than Ori-cell (*p* < 0.0001, Student’s t test, Figures 3A and 3B; Figures S6 and S7; Table S8). Then, western blot was applied to quantitatively measure the expression level of EGFP in HEK293T cells. The protein expression level of EGFP in Opt-cell was approximately twice that in Ori-cell (*p* < 0.001, Student’s t test, Figures 3C and 3D; Figure S9) consistent with the fluorescence intensity analysis. To clarify whether the difference in EGFP protein expression levels was due to different transfection efficiencies or differences in transcriptional levels, we adopted real-time quantitative PCR to evaluate the EGFP mRNA content in each group of cells. The results showed that there was no significant difference in mRNA expression levels between Ori-cell and Opt-cell (Figure 3E; Table S9), indicating that the difference in EGFP expression was mainly due to differences at the translational level.

Finally, we attempted to optimize sequences other than EGFP with UTailoR to validate its value in practical applications. We carried out the optimization procedure on the UTR sequence of hepatitis B virus core antigen and compared the translation efficiency of the original sequence and the optimized sequence in HEK293T cells. Moreover, we examined the translation efficiency after replacing the original UTR sequence with the human alpha globin UTR sequence, which is a prevalently employed UTR optimization approach at present. The results demonstrated that, in the case of no significant difference in transfection efficiency (Figure S10), the translation efficiency of the optimized group was more than twice that of the original group (Figure 3F; Figure S11). Notably, compared with the human alpha globin UTR sequence, the translation efficiency of the optimized group also increased by approximately 40% (Figure 3G). This part of results support that UTailoR can be generalized to common sequences and its effect is superior to the currently widely utilized UTR optimization methods.

In summary, we first announced that the UTR sequences optimized by UTailoR exhibit higher translation efficiency than the original sequences. Importantly, the improvement in translation efficiency is greater than that of the commonly used universal UTR at present. This further confirmed the reliability of the calculation results of our model.

### Develop the online tool for optimizing 5′ UTR sequences

**Figure 4:**
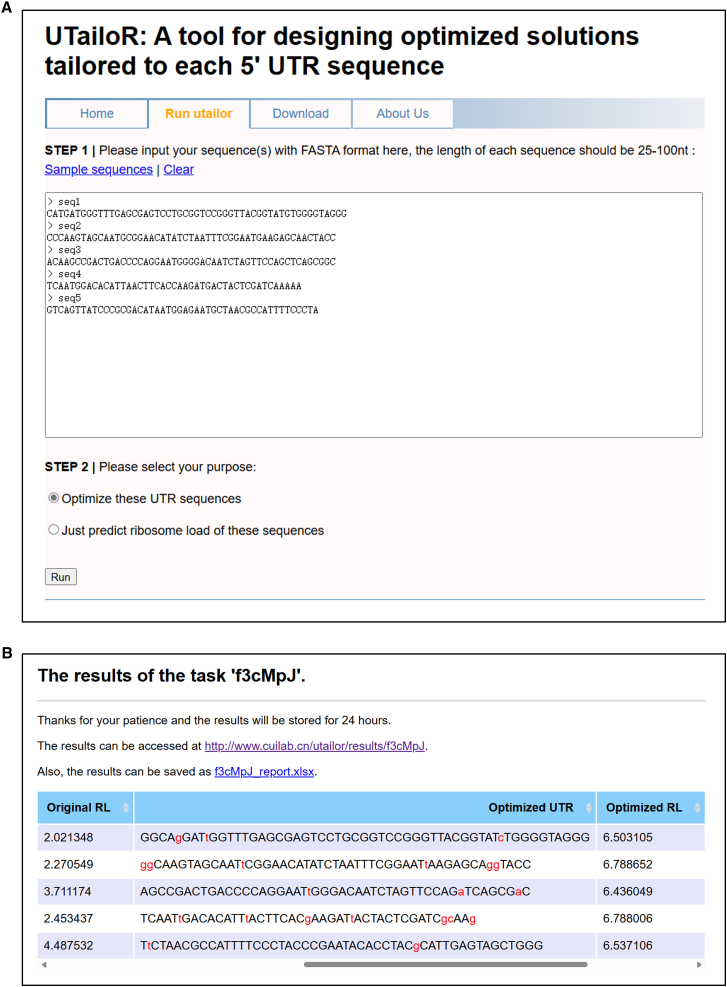
Overview of the online tool (A) The main program page of UTailoR. The program accepts FASTA format input and can be used to predict the MRL score of input sequences or generate optimized sequences. (B) The results display the page of UTailoR. The output results can be browsed online or downloaded as xlsx format.

In order to make the UTailoR algorithm easier to apply, we have developed an online tool, which can be freely accessed at http://www.cuilab.cn/utailor. UTailoR accepts 5′ UTR sequences with lengths ranging from 25 to 100 nt as input, first predicts their translation efficiency, and then devises a unique optimization scheme for each sequence (Figure 4A). For the 5 example 5′ UTR sequences, the entire process takes less than 30 s, rendering it more convenient and efficient compared to genetic algorithms or other deep-learning-based methods.^7^^,^^21^^,^^31^ The results included the original sequence, the optimized sequence, and their respective MRL scores (Figure 4B). We utilized both fixed-length (50 nt) and variable-length (25–100 nt) datasets to train two discriminative prediction models in order to enhance result accuracy. For the fixed-length model, input sequences longer than 50 nt will be processed using the last 50 nt, whereas those shorter than 50 nt will have padding added on the left side, which was similar to the method we handle sequences for the variable-length model.

## Discussion

In this study, we introduce an innovative 5′ UTR sequence optimization strategy for mRNA-based therapeutics. In contrast to conventional approaches, our method tailors individualized optimization schemes for each UTR sequence, offering enhanced flexibility while preserving the original sequence features to mitigate potential adverse effects of excessive modifications to the UTR. This advancement is enabled by deep learning technology, which not only validates existing human knowledge^8^^,^^10^ but also captures previously unnoticed patterns. We anticipate that insights gained from deep learning will further advance our understanding of the role of the 5′ UTR in translation efficiency.

Regarding the 5′ UTR sequence optimization issue, the uORF theory^9^ and Kozak sequence^32^ are representative achievements of human knowledge. We discovered that the output results of UTailoR in the test set were completely devoid of uORFs, which is in accordance with our understanding. Similarly, in the sequence preferences exhibited by UTailoR, the A at the third position upstream of the start codon (3-A) was strongly preferred, which aligns with the characteristics of the Kozak sequence. Nevertheless, in the optimized outcomes, it was challenging to identify typical Kozak sequences. This implies that the Kozak sequence is not necessarily the sole solution, and customizing unique UTRs for each sequence can achieve better results. This serves as an illustration of how deep learning methods surpass human knowledge.

From computational standpoint, UTailoR is capable of optimizing 5′ UTR sequences within 100 nt only. According to the research by Sample et al.,^16^ this length merely covers 29% of human 5′ UTRs. However, on the one hand, 5′ UTR sequences in bacterial and viral genomes are relatively shorter, resulting in more UTR sequences being covered. On the other hand, existing studies have confirmed that the region near the start codon of 5′ UTRs has a significant impact on translation efficiency.^33^^,^^34^ Therefore, for UTR sequences longer than 100 nt, optimizing the 100 nt sequence upstream of the start codon is a reasonable and effective solution. In summary, we are convinced that UTailoR is sufficient to solve the majority of 5′ UTR optimization problems.

Although UTailoR has achieved the state-of-the-art performance, our research still possesses limitations. One unresolved matter is how to understand the optimization process of UTailoR. The MPRA dataset reveals a series of 5′ UTR sequences with high translation efficiency, but it seems difficult to find commonalities among them. Although AI-based methods demonstrate their powerful feature extraction capabilities, like many deep learning methods, these patterns are difficult for humans to interpret. In this paper, we merely discussed the characteristics of individual nucleotides. How specific patterns composed of multiple nucleotides affect translation efficiency remains to be further explored.

Another issue is that UTailoR only optimizes 5′ UTR sequences and does not consider the properties of the CDS region and 3′ UTR. Currently, the optimization methods for the CDS region are relatively mature, and UTailoR can be utilized concurrently with these methods. Regarding the 3′ UTR, there is currently limited research. Based on the current understanding of the function of 3′ UTRs, predicting the microRNA-binding sites in 3′ UTRs might be a feasible solution. Simultaneously, in addition to the independent effects of each component of mRNA on translation efficiency, the overall interaction of the full-length mRNA sequence is also worth considering, for instance, whether the full-length mRNA forms more complex secondary structures and how different secondary structures affect translation efficiency and stability, etc. Although it is easy to capture the correlation between 5′ UTR sequences and translation efficiency through the MPRA dataset, the interaction effects between 5′ UTRs and other parts are difficult to quantify. Furthermore, under physiological conditions, mRNA undergoes various modifications, many of which have been verified to influence translation efficiency.^35^ Incorporating these modifications of mRNA into the prediction of translation efficiency and mRNA optimization strategies is a direction worthy of further investigation.

In summary, due to the current lack of data on the influence of full-length 5′ UTR on translation efficiency, it is difficult to optimize the full-length range of 5′ UTR through deep learning methods. Previous studies have attempted to optimize the exogenous UTR based on the human genome UTR,^36^ which is similar to the idea of optimizing the CDS region. However, a prominent issue is that the CDS region has species-specific characteristics due to codon bias,^6^^,^^37^ while there is no similar theoretical support for the UTR. We look forward to the emergence of more comprehensive high-quality datasets in the future, allowing for more in-depth research on these issues.

### Limitations of the study

In this study, we propose a deep learning approach to optimize the mRNA 5′ UTR sequence and validate its efficacy through cellular experiments. However, a primary limitation lies in the fact that the investigation focused solely on the impact of the 5′ UTR sequence itself on translation efficiency, without considering its potential interactions with the CDS and the 3′ UTR. Furthermore, there remains a lack of intuitive interpretation for the features identified by the deep learning model that contribute to sequences with high translation efficiency.

## Resource availability

### Lead contact

Requests for further information and resources should be directed to and will be fulfilled by the lead contact, Qinghua Cui (cuiqinghua@bjmu.edu.cn).

### Materials availability

This study did not generate new unique reagents.

### Data and code availability

• This article analyzes existing, publicly available data, accessible at https://doi.org/10.1038/s41587-019-0164-5, https://doi.org/10.1101/gr.224964.117, https://doi.org/10.1038/s41467-021-24436-7, and https://doi.org/10.1016/j.cels.2023.10.011.
• All data supporting the findings of this study are available within the article and its supplemental information.
• All original code has been deposited at http://www.cuilab.cn/utailor/download and is publicly available as of the date of publication.
• Any additional information required to reanalyze the data reported in this article is available from the lead contact upon request.

## Acknowledgments

This study was supported by grants from the 10.13039/501100001809National Natural Science Foundation of China (62025102 and 81921001) and the Scientific and Technological Research Project of 10.13039/501100009967Xinjiang Production and Construction Corps (2023AB057, 2023ZD037, and 2022ZD001).

## Author contributions

Q.C. and L.L. supervised the study. Y.L. performed the study. C.C. helped to develop and debug the UTailoR Web server. Y.L. wrote the raw manuscript. Q.C., L.L., C.C., and Y.L. edited the manuscript.

## Declaration of interests

The authors declare no competing interests.

## STAR★Methods

### Key resources table

**Table:** 

REAGENT or RESOURCE	SOURCE	IDENTIFIER
**Antibodies**		

eGFP Monoclonal Antibody	Thermo	Cat# F56-6A1.2.3; RRID: AB_889471
FLAG tag Mouse monoclonal antibody	Biodragon	Cat# B1084
Actin beta Mouse Monoclonal Antibody	Biodragon	Cat# B1029; RRID: AB_3713074
HRP-goat anti mouse IgG	Biodragon	Cat# BF03001; RRID: AB_3105782

**Chemicals, peptides, and recombinant proteins**		

Endotoxin free plasmid small extract medium kit	TIANGEN	Cat# DP118
RNA Easy Fast Animal tissue/cell total RNA Extraction Kit	TIANGEN	Cat# DP451
DMEM Medium	Solarbio	Cat# 11995
Trypsin-EDTA solution, 0.25%	Solarbio	Cat# T1300
BCA Protein Assay Kit	Solarbio	Cat# PC0020
Penicillin-Streptomycin Liquid	Solarbio	Cat# P1400
Precast SDS-PAGE Gel 15%	Solarbio	Cat# PG01510-S
RIPA Buffer	Solarbio	Cat# R0010
Fetal Bovine Serum	Gibco	Cat# A5670701
Opti-MEM™ Medium	Gibco	Cat# 31985070
Lipofectamine™ 3000 transfection reagent	Invitrogen	Cat# L3000015
HiScript III All-in-one RT SuperMix	Vazyme	Cat# R333
Taq Pro Universal SYBR qPCR Master Mix	Vazyme	Cat# Q712

**Deposited data**		

HEK293T cell MPRA dataset	Sample et al.^16^	
S. cerevisiae MPRA dataset	Cuperus et al.^28^	
Human Ribo-seq dataset	Cao et al.^12^	
Human FACS-seq dataset	Zheng et al.^17^	

**Experimental models: Cell lines**		

HEK 293T	Peking University	N/A
Hela	Beyotime	Cat# C6330
HUVECs	Freemore	Cat# 200-0630

**Oligonucleotides**		

h-β-actin-F-5′-TAAGGAGAAGCTGTGCTACGTC-3′	TsingkeBiotecnology	N/A
h-β-actin-R-5′-TTTCGTGGATGCCACAGGAC-3′	TsingkeBiotecnology	N/A
h-EGFP-F-5′-CTACCCCGACCACATGAAGC-3′	TsingkeBiotecnology	N/A
h-EGFP-R-5′-CTTGTAGTTGCCGTCGTCCT-3′	TsingkeBiotecnology	N/A

**Recombinant DNA**		

Ori-1-EGFP overexpression plasmid	HanBio	N/A
Ori-2-EGFP overexpression plasmid	HanBio	N/A
Ori-3-EGFP overexpression plasmid	HanBio	N/A
Opt-1-EGFP overexpression plasmid	HanBio	N/A
Opt-2-EGFP overexpression plasmid	HanBio	N/A
Opt-3-EGFP overexpression plasmid	HanBio	N/A
HBV-ori overexpression plasmid	HanBio	N/A
HBV-opt overexpression plasmid	HanBio	N/A
HBV-HbA overexpression plasmid	HanBio	N/A

**Software and algorithms**		

Python 3.10	N/A	https://www.python.org
UTailoR	This Paper	http://www.cuilab.cn/utailor/download
Graphpad Prism 9.5.1	GraphPad Prism Software, Inc	https://www.graphpad.com/features
ImageJ	National Institutes of Health	https://imagej.net/ij

### Experimental model and study participant details

#### Cell culture

The HEK293T cell**s**, HUVECs, and HeLa cell lines used in this study were cultured in high-glucose DMEM **(Cat# 11995)** medium supplemented with 10% FBS (Cat# A5670701) and 1× penicillin/streptomycin (Cat# P1400). The cells were maintained at 37 °C in a humidified incubator with 5% CO_2_ and were passaged when the confluence reached 70%–80%. Mycoplasma contamination testing was performed every six months. All cell lines were accompanied by an authentication report provided by the respective supplier.

### Method details

#### Datasets and data processing

We collected the following three types of datasets (Table 2) in this study.

(1) The MPRA dataset, which includes the data from human 293T cell line published by Sample et al.^16^ and the yeast dataset published by Cuperus et al.^28^ Sample et al. used the Mean Ribosome loading (MRL) to represent the translation efficiency, while Cuperus et al. represented the translation efficiency by the growth rate of yeast.
(2) The Ribo-seq dataset, consisting of data from the 293T cell line, PC3 cell line, and muscle tissue dataset collected by Cao et al.,^12^ represents translation efficiency through the ratio of Ribo-seq RPKM to RNA-seq RPKM. Following Cao et al.'s approach, we excluded sequences with low Ribo-Seq RPKM or RNA-seq RPKM to ensure sequencing result reliability.
(3) The FACS-seq dataset, which includes the data form 293T and ES cells published by Zheng et al.^17^ Zheng et al. used fluorescence intensity to represent translation efficiency, and classified the top 5% of UTR sequences as positive samples and the bottom 5% as negative samples.

We used one-hot encoding with a total of 5 bits to represent each base, representing A, T, C, G, and pad. For sequences shorter than 100 nt, we applied left-side padding to extend them to 100 nt; and for sequences longer than 100 nt, we select the last 100 nt for encoding. In summary, all UTR sequences can be depicted as a 2D array with shape (100, 5).

#### Constructing the discriminative model

We constructed a deep learning model using the functional API of TensorFlow 2.11. The discriminative model takes a sequence feature with a shape of (100,5) as input, which is then passed through three residual-connected convolutional layers, followed by a gate recurrent unit (GRU) layer and three residual-connected dense layers. Finally, it outputs the predicted MRL score for the sequence. The hyperparameters of the neural network were determined using HyperBand optimization technique.

To train the model, we utilized the dataset published by Sample et al. Initially, 10% of the data was randomly selected as the test set while the remaining data was divided into an 80:20 ratio to create training and validation sets, respectively. The mean squared error was employed as the loss metric for evaluating the model’s performance. The initial learning rate was set to 0.001 during training process. If there was no reduction in validation set loss for 5 consecutive epochs, we adjusted the learning rate to 1/10 of its original value; furthermore, if there was no further reduction in loss after 12 epochs, we terminated training to prevent overfitting (Figure S1).

Subsequently, we applied Model Soups method to optimize the model’s weights.^38^ Specifically, we randomly partitioned the training set and validation set, repetitively trained 10 models with identical structures but different weights. These models were then ranked based on their performance on the test set and finally averaged their weights using a greedy algorithm to obtain final weights for our model.

#### Optimizing hyperparameters

We used HyperBand to optimize hyperparameters.^39^ Initially, 8000 groups of hyperparameters were randomly selected in the hyperparameter space. For each group of hyperparameters, we trained 2 epochs, and then drop 2/3 hyperparameter groups with poor performance. The remaining hyperparameter groups will be trained 4 more epochs, and then drop 2/3 hyperparameter groups again, training 8 more epochs for remaining hyperparameter groups. This procedure was performed until the optimal combination of hyperparameters was obtained. The hyperparameter space includes the number of convolution kernels, the size of convolution kernels, the output dimension of GRU, the number of fully connected layers, the number of neurons in each fully connected layer, and the activation function.

#### Baseline methods

To evaluate the performance of the discriminative model, we conducted comparative analysis with previously proposed methods as presented in Table 1. Specifically, we re-implemented Optimus 5-Prime^16^ in TensorFlow 2.11 according to the description provided by Sample et al. For FramePool^20^ and MTtrans,^17^ we used the pre-compiled model files provided by their respective authors. As for the 5′ UTR LM,^21^ we obtained the foundational model and appended a fully connected layer to generate predicted MRL scores. Notably, all models were retrained on the variable-length MPRA dataset (with only training of the appended fully connected layer for 5′ UTR LM).

#### Interpretability of the discriminative model

We used SHapley Additive exPlanations (SHAP) to evaluate the importance of input features.^40^ SHAP is developed on the basis of game theory, which calculates the marginal contribution of each feature by introducing each feature in a different order. Through the marginal contribution, we can get to what extend and how each feature influence on the results, as shown in Equation 1:

(Equation 1) $$\text{SHAP}\left(x\right)=\sum_{f=1}^{n}\frac{y_{x\in setf}-y_{x\notin setf}}{f\times \left(\begin{array}{l} f\\ n \end{array}\right)}$$

Where “SHAP(x)” represents the SHAP value of feature x, “*n*” is the total number of features, “*f*” represents the rank of features *x* introduced into the model, $y_{x\in setf}$ and $y_{x\notin setf}$ represent the predicted value of the model when feature *x* is included or not included in the feature set, respectively.

#### Constructing the generative model

The generative model was constructed based on an auto-encoder architecture. It takes a sequence feature of shape (100, 5) as input and passes it through three convolutional layers and one fully connected layer to generate a latent vector of length 128. Subsequently, the latent vector was reconstructed into a sequence feature with the same shape using a fully connected layer and three deconvolutional layers. To make the model have generation capability, we have devised a unique loss function for the model comprising two components: reconstruction loss (Equation 2) and RL loss (Equation 3).

(Equation 2) $$RE\_loss=CCE\left(y_{\text{true}},y_{\text{pred}}\right)+BCE\left(y_{\text{true}}\left[-1\right],y_{\text{pred}}\left[-1\right]\right)$$

(Equation 3) $$RL\_loss=e^{MRL\left(y_{\text{true}}\right)-MRL\left(y_{\text{pred}}\right)}$$

Where CCE denotes the categorical cross-entropy between the reconstructed vector and the original vector, while BCE denotes the binary cross-entropy between the last element of the reconstructed vector and the last element of the original vector, which represents ‘pad’. In RL loss, MRL(y) refers to the predicted MRL score of vector *y* by our prediction model. The final form of our loss function is given by Equation 4:

(Equation 4) Total_loss=RE_loss+λ·RL_loss

Where λ serves as a hyperparameter for adjusting weights associated with both parts of losses; based on preliminary experiments, we set λ = 100.

#### Plasmid construction

Select the top 3 pairs of 5′ UTR sequences with the most increased MRL scores from the test set and commission Hanbio Biotechnology to synthesize the corresponding DNA fragments. Each DNA fragment consists of a 25bp linker sequence, a 50bp 5' UTR sequence, and a 720bp EGFP CDS, resulting in a total length of 795bp (Table S1). For the hepatitis B virus core antigen, the sequence is likewise composed of a variable 5′ UTR and a consistent CDS region. The length of the final inserted sequence is 784 bp or 765 bp, since the length of the HbA-UTR sequence is not in accordance with that of the original UTR sequence of the hepatitis B virus core antigen (Table S2). We did not undertake any artificial design for the 3’ UTR sequence. This implies that all the RNA sequence after transcription would have a uniform 3′ UTR sequence, namely the sequence ranging from the multiple cloning site on the pcDNA3.1 plasmid to the poly A region. Utilize restriction enzyme digestion to insert the target fragments into the multiple cloning site of the pcDNA3.1 vector, then transform DH5α competent cells, and select single clones after culturing for 12 h. After an additional incubation period of 18 h, collect bacterial suspension and perform plasmid extraction according to the protocol provided in the TIANGEN plasmid extraction kit (Cat# DP118).

#### Cell transfection

Inoculate 2×10^6^ HEK293T cells into 10 cm cell culture dishes and incubate them in DMEM complete culture medium for 48 h. Following the recommended dosage in the Lipo3000 transfection reagent (Cat# L3000015) protocol, transfect 10 μg of the corresponding plasmid into each dish of cells. After 24 h, evaluate the fluorescence intensity using a fluorescence microscope and centrifugate cells. For human umbilical vein endothelial cells (HUVECs) and human cervical cancer HeLa cells, we followed the same culture conditions as for HEK293T cells, but fluorescence intensity was measured 36 h after transfection.

#### Relative quantification of EGFP and HBcAg

We divided the collected cells evenly into two parts, which will be used to extract mRNA and proteins respectively. mRNA was extracted using the TIANGEN RNA extraction kit (Cat# DP451). Take 1 μg of mRNA to perform reverse transcription using Vazyme All-in-one RT SuperMix (Cat# R333), and then dilute the resulting cDNA product at a ratio of 1:10. Vazyme Taq Pro Universal SYBR qPCR Master Mix (Cat# Q712) with primers listed in Table 1 was used for fluorescence quantitative PCR.

Total proteins were extracted from cells using Solabio RIPA lysis buffer (Cat# R0010), and the protein concentration was adjusted to 1 μg/ul using the BCA method for Western Blot sample preparation. Electrophoresis was performed using Solabio 15% Precast-Gel (Cat# PG01510) with 10 μl of sample per well. The PVDF membrane underwent sequential incubation with primary antibody against EGFP (Cat# F56-6A1.2.3) and HRP-conjugated goat anti-mouse IgG before being placed on the Bio-rad ChemiDoc XRS+ Chemiluminescence Imaging System for chemiluminescence detection. ImageJ software was utilized for quantitative analysis of protein blot. For the quantification of HBcAg, we followed a similar approach using the FLAG tag Mouse Monoclonal Antibody (Cat# B1084) and Actin beta Mouse Monoclonal Antibody (Cat# B1029).

### Quantification and statistical analysis

The data are displayed as Mean ± SEM in other experimental data. ∗*p* < 0.05, ∗∗*p* < 0.01, ∗∗∗*p* < 0.001 and ∗∗∗∗*p* < 0.0001 were used to determine the statistical significance of differences. Differences among the groups were analyzed using Student’s t test or one-way ANOVA for multiple comparisons with Bartlett’s test. All statistical analyses were performed with Scipy and Graphpad Prism 9.5.1. Visualization of computational experiment results was performed using the Matplotlib and Seaborn libraries in Python, while biochemical experiment results were visualized using Graphpad Prism. The web server was built based on Django 4.2.

## Supplemental information

Document S1. Figures S1–S11 and Tables S1–S3, S7, and S9

Table S4. Optimization results of the 5′ UTR generation model on the test set sequences (50 nt), related to Figure 2

Table S5. Optimization results of the 5′ UTR generation model on the test set sequences (25–100 nt), related to Figure 2

Table S6. Performance of the generative model with different λ, related to Figure 2

Table S8. Raw data of fluorescence intensity analysis, related to Figure 3
