# Supplementary material for: Enhancing mRNA translation efficiency with discriminative and generative artificial intelligence by optimizing 5′ UTR sequences
Source: iScience. 2025 Sep 10;28(10):113544. doi: 10.1016/j.isci.2025.113544 (PMC12506572; doi:10.1016/j.isci.2025.113544)
Supplement: Document S1. Figures S1–S11 and Tables S1–S3, S7, and S9 [file mmc1.pdf]

iScience, Volume *28*

Supplemental information

Enhancing mRNA translation efficiency with discriminative and generative artificial intelligence by optimizing 5′ UTR sequences

Yu Liu, Chunmei Cui, Limei Liu, and Qinghua Cui

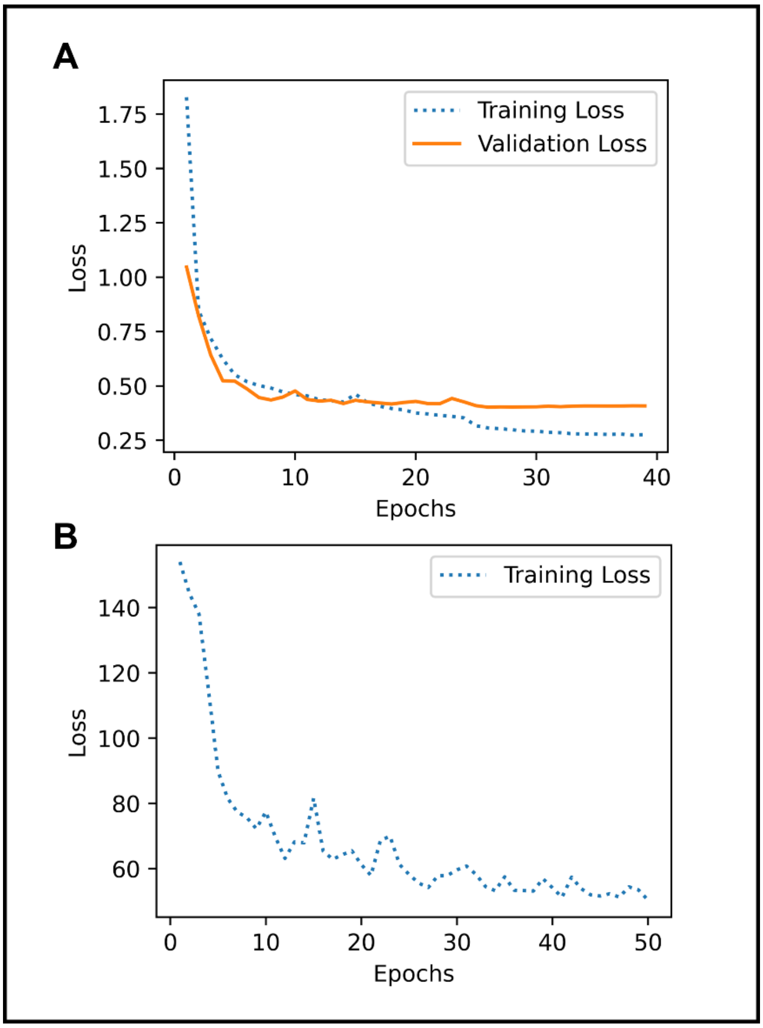

Figure S1. The loss decay curves for the discriminative model and the generative model during training. (A) represents the discriminative model, and (B) represents the generative model.

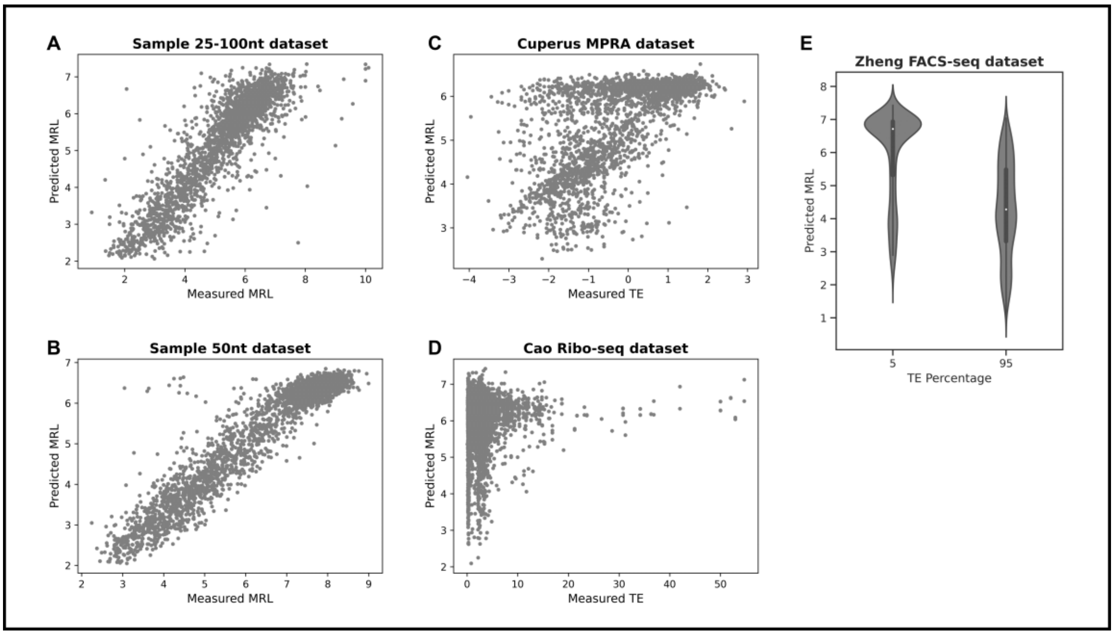

Figure S2. Performance of the discriminative model across various datasets. **(A)-(D)** Scatter plots illustrating the correlation between predicted values and actual translation efficiency, with dataset names provided as titles. **(E)** Violin plot presenting the disparity in predicted translation efficiency values between sequences ranked in the top 5% and bottom 5% on the FACS-seq classification dataset.

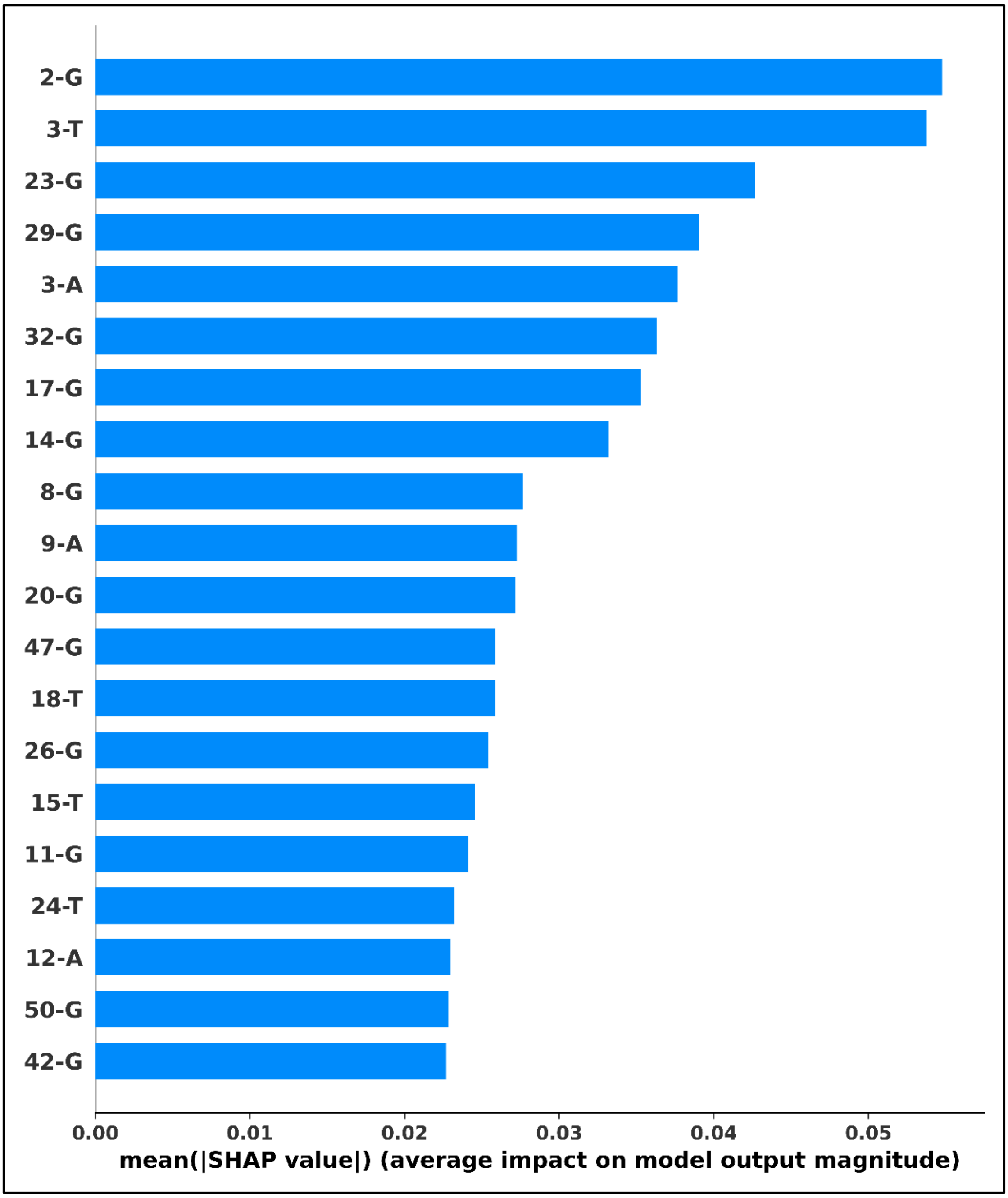

Figure S3. The top 20 most important features for the discriminative model. The feature importance is sorted in descending order based on the absolute value of SHAP.

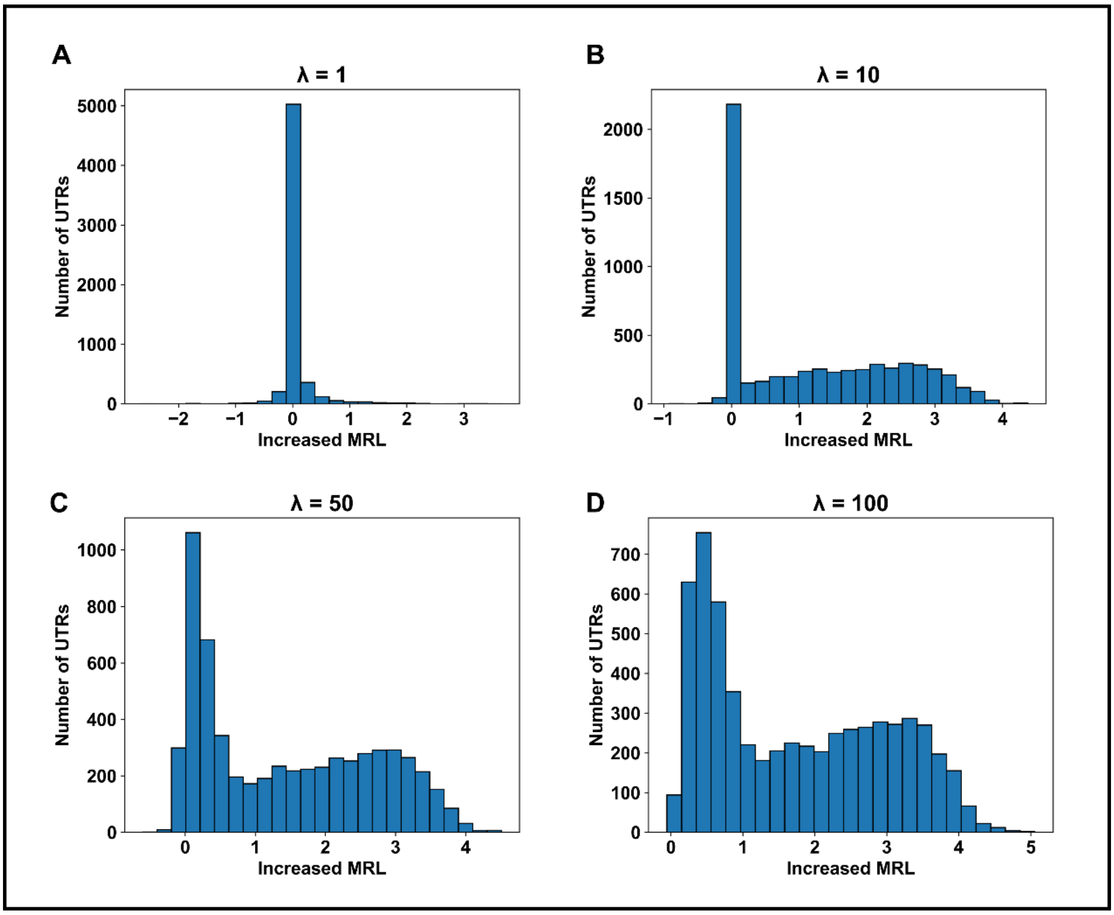

Figure S4. The histogram of the increase in MRL score for the optimized sequences compared to the original sequences, obtained by taking different λ values during training of the generative model.

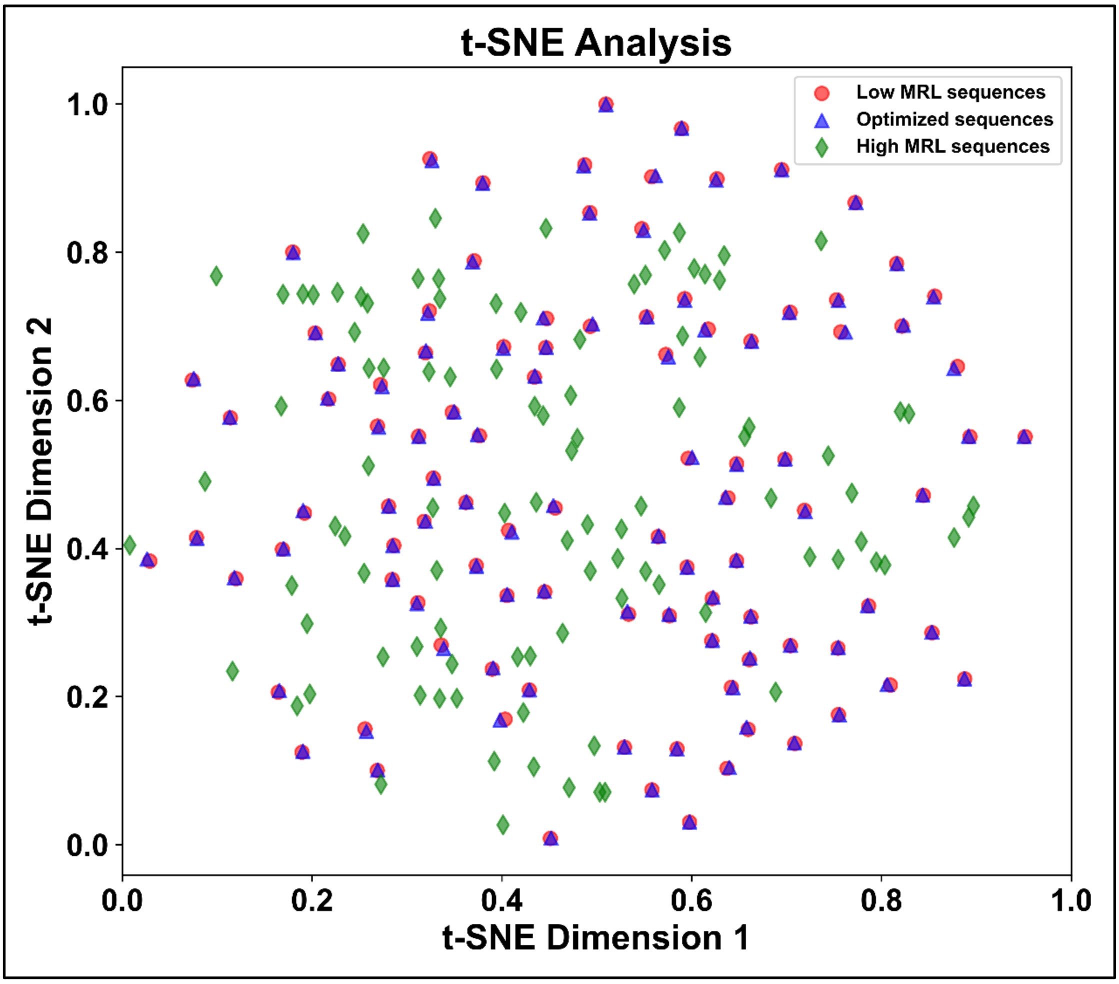

Figure S5. Distribution of the original sequences, optimized sequences, and known high translation efficiency sequences in the t-SNE space. The sample size of each kind of sequences is 100.

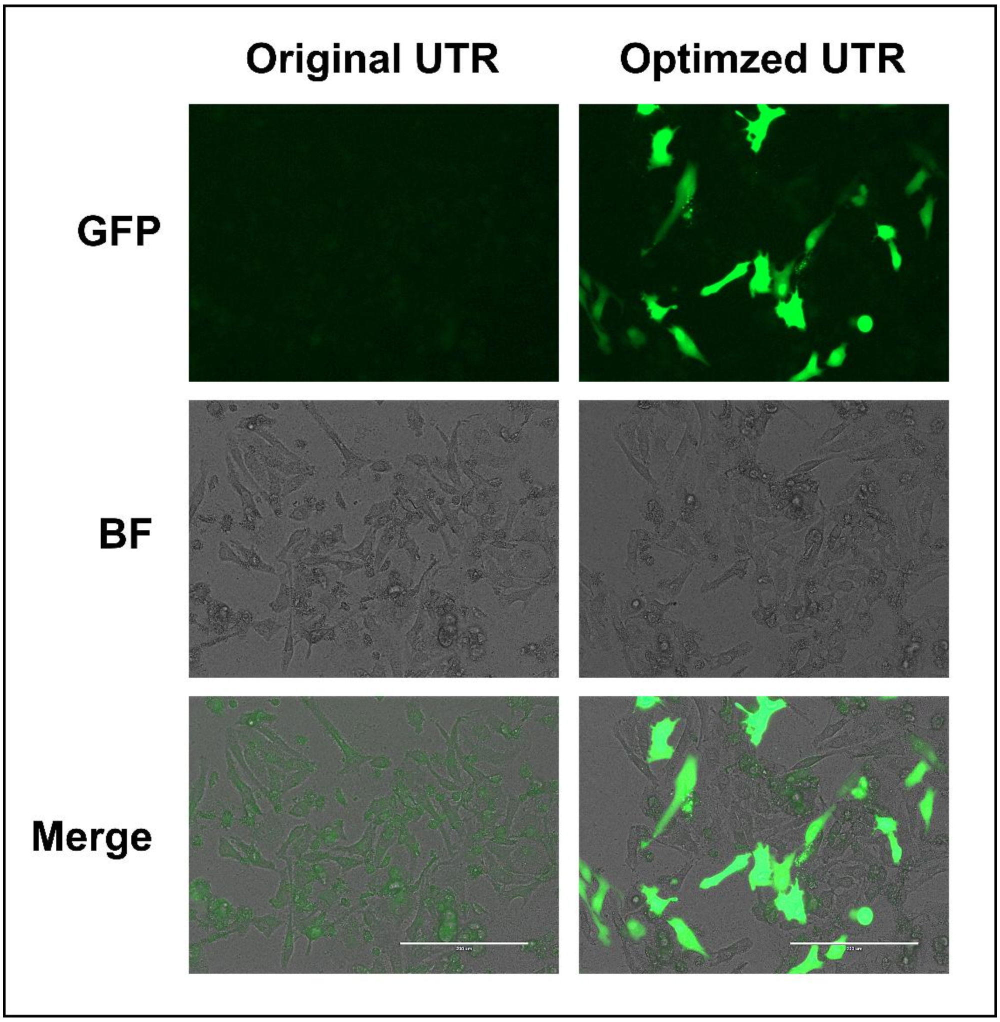

Figure S6. Fluorescence microscopy images of Hela cells, taken 36 hours after transfection, magnified 400x. BF: bright field.

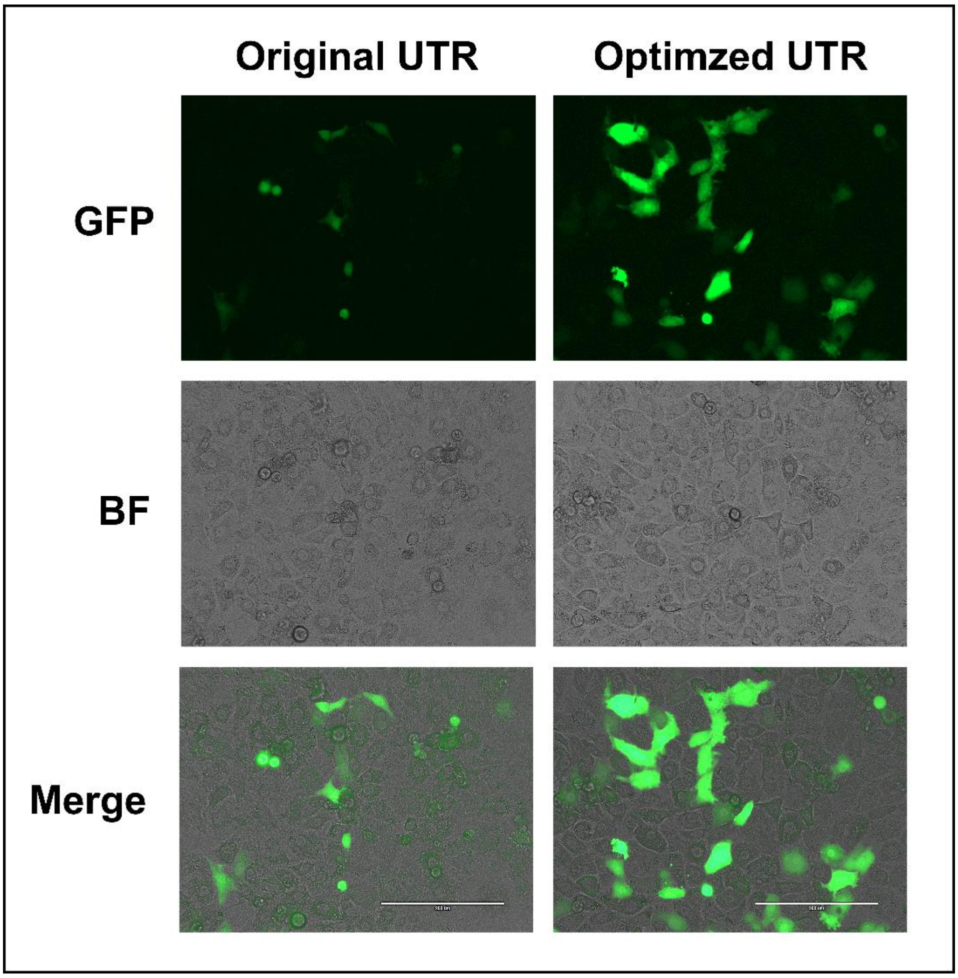

Figure S7. Fluorescence microscopy images of HUVECs, taken 36 hours after transfection, magnified 400x. BF: bright field.

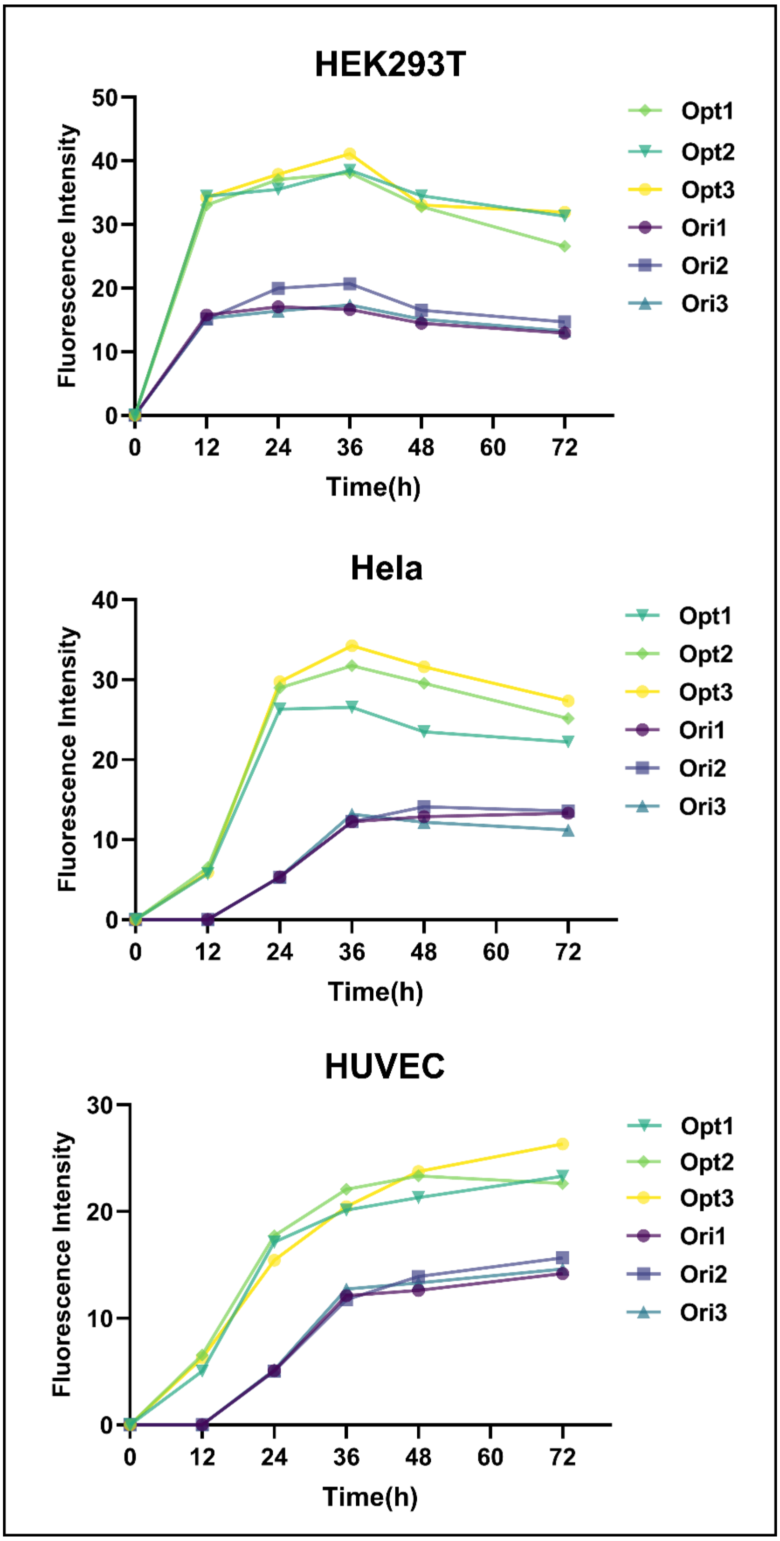

Figure S8. The fluorescence intensity changes of three types of cells over time. Five fields of view were taken from each sample at each time point for statistical analysis.

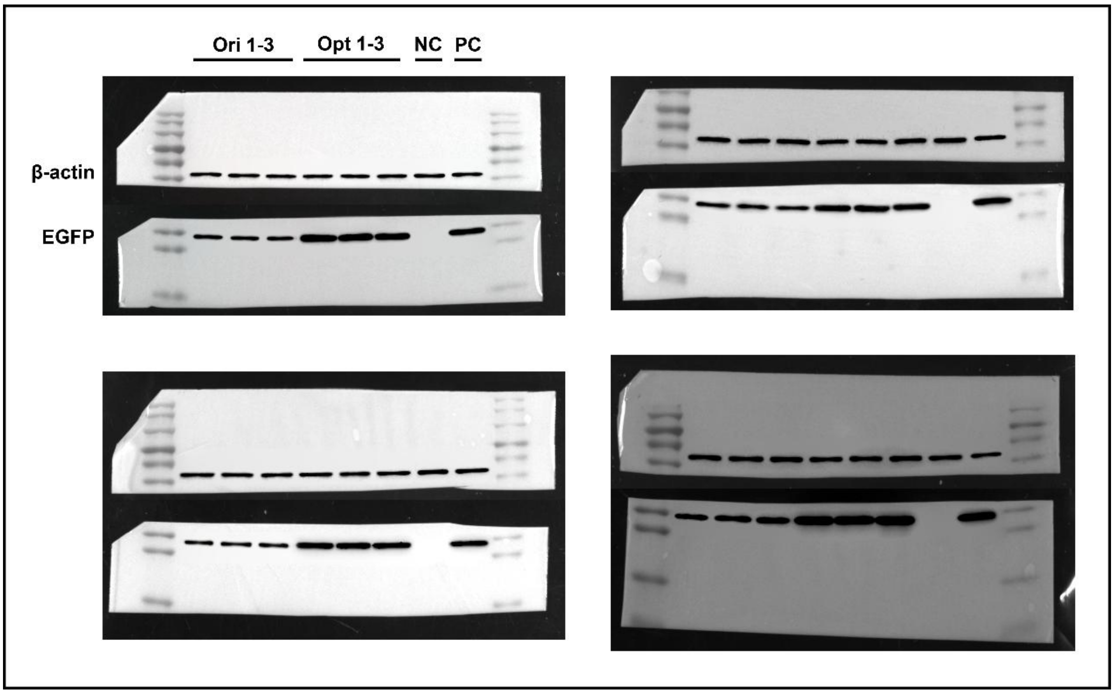

Figure S9. The original images of Western Blot for detecting EGFP expression. The PVDF membrane was sectioned between the markers of 35kDa and 40kDa for separate incubation with the antibody against β-actin and the antibody against EGFP.

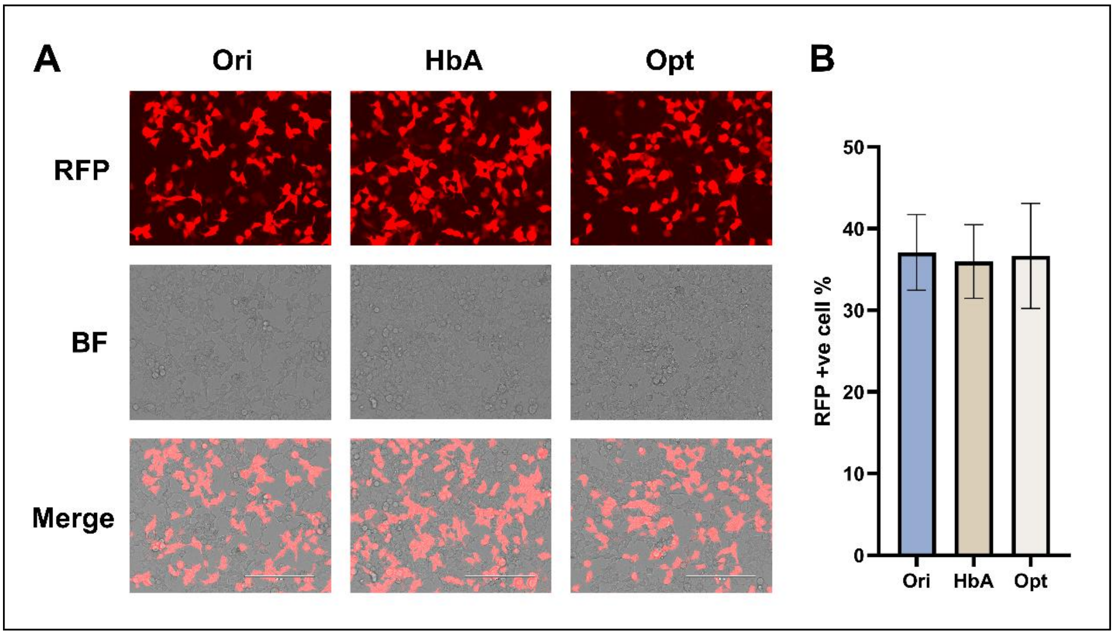

Figure S10. Evaluating the transfection efficiency of HBcAg plasmid by red fluorescent protein (RFP). **(A)** Fluorescence microscopy images of HEK293T cell, taken 36 hours after transfection, magnified 400x. BF: bright field. **(B)** Statistical analysis of the proportion of RFP expressing cells, with 3 fields of view taken from each sample and 4 samples per group.

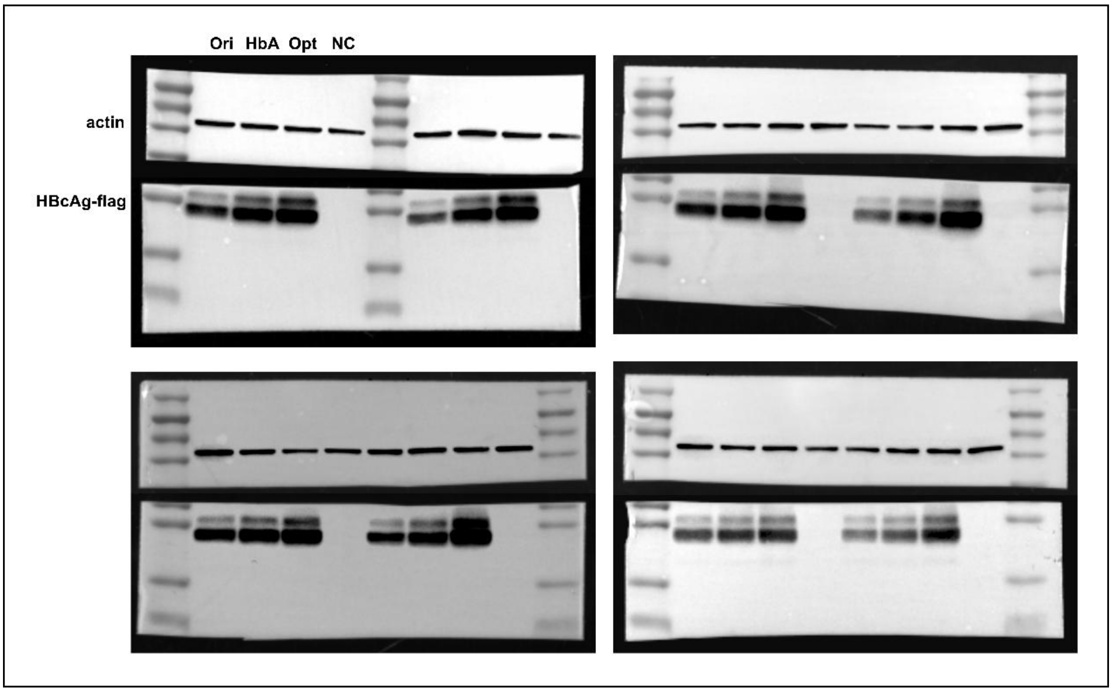

Figure S11. The original imagines of Western Blot for HBcAg test. The PVDF membrane was sectioned between the markers of 35kDa and 40kDa for separate incubation with the antibody against β-actin or the antibody against flag.

Table S1. Original 5' UTR and optimized 5' UTR of EGFP sequence

**Linker**	GGGACATCGTAGAGAGTCGTACTTA
**Ori1**	ATTAGAAATGGATGTTTGTGACCGTAATATTGTGGCGACGCCGTAC<br>TGGA
**Opt1**	ATTAGAAATAGTTGTTTGTGACCGTAATATTGTGGCGAAGCAGTAC<br>TGGA
**Ori2**	CCGGCGGAAAATGCCAGAGTTCGGGTGGAAAGATAACGAAAGGCG<br>ATGCC
**Opt2**	CCGGAGGAAATTGCCAGAGTTCGAGTAGAAAGATAACGAAAGGCG<br>TTGAC
**Ori3**	TCGTAAGATGGCTTATTCGTACAGGACGGGTTCGGAATCCGGCTAT<br>TCGA
**Opt3**	TCGTAAGATAGCTTATTCGTATAGGACGGGTTCGGAATCCGGCTAT<br>TAGA
**EGFP**	ATGGTGAGCAAGGGCGAGGAGCTGTTCACCGGGGTGGTGCCCATC<br>CTGGTCGAGCTGGACGGCGACGTAAACGGcCACAAGTTCAGCGTGT<br>CCGGCGAGGGCGAGGGCGATGCCACCTACGGCAAGCTGACCCTGA<br>AGTTCATCTGCACCACCGGCAAGCTGCCCGTGCCCTGGCCCACCCT<br>CGTGACCACCCTGACCTACGGCGTGCAGTGCTTCAGCCGCTACCCC<br>GACCACATGAAGCAGCACGACTTCTTCAAGTCCGCCATGCCCGAAG<br>GCTACGTCCAGGAGCGCACCATCTTCTTCAAGGACGACGGCAACTA<br>CAAGACCCGCGCCGAGGTGAAGTTCGAGGGCGACACCCTGGTGAA<br>CCGCATCGAGCTGAAGGGCATCGACTTCAAGGAGGACGGCAACAT<br>CCTGGGGCACAAGCTGGAGTACAACTACAACAGCCACAACGTCTA<br>TATCATGGCCGACAAGCAGAAGAACGGCATCAAGGTGAACTTCAA<br>GATCCGCCACAACATCGAGGACGGCAGCGTGCAGCTCGCCGACCA<br>CTACCAGCAGAACACCCCCATCGGCGACGGCCCCGTGCTGCTGCCC<br>GACAACCACTACCTGAGCACCCAGTCCGCCCTGAGCAAAGACCCC<br>AACGAGAAGCGCGATCACATGGTCCTGCTGGAGTTCGTGACCGCC<br>GCCGGGATCACTCACGGCATGGACGAGCTGTACAAGTAA

Table S2. Original 5' UTR, optimized 5' UTR and HbA UTR of HBcAg sequence

**Ori-UTR**	TGGGGGAGGAGATTAGGTTAAAGGTCTTTGTACTAGGAGGC<br>TGTAGGCATAAATTGGTCTGCGCACCAGCACC
**Opt-UTR**	TTGGGGAGGAGATTAGGTTAAACGTCTTTGTACTAGGAGGCT<br>GTAGGCATAAATTGGTCTGGGCACGGGCACG
**HbA-UTR**	GAGAATAAACTAGTATTCTTCTGGTCCCCACAGACTCAGAGA<br>GAACCCGCCACC
**HBV-flag CDS**	ATGCAACTTTTTCACCTCTGCCTAATCATCTCTTGTTCATGTC<br>CTACTGTTCAAGCCTCCAAGCTGTGCCTTGGGTGGCTTTGGG<br>GCATGGACATCGACCCTTATAAAGAATTTGGAGCTACTGTGG<br>AGTTACTCTCGTTTTTGCCTTCTGACTTCTTTCCTTCAGTACG<br>AGATCTTCTAGATACCGCCTCAGCTCTGTATCGGGAAGCCTT<br>AGAGTCTCCTGAGCATTGTTCACCTCACCATACTGCACTCAG<br>GCAAGCAATTCTTTGCTGGGGGGAACTAATGACTCTAGCTAC<br>CTGGGTGGGTGTTAATTTGGAAGATCCAGCGTCTAGAGACCT<br>AGTAGTCAGTTATGTCAACACTAATATGGGCCTAAAGTTCAG<br>GCAACTCTTGTGGTTTCACATTTCTTGTCTCACTTTTGGAAGA<br>GAAACAGTTATAGAGTATTTGGTGTCTTTCGGAGTGTGGATT<br>CGCACTCCTCCAGCTTATAGACCACCAAATGCCCCTATCCTA<br>TCAACACTTCCGGAGACTACTGTTGTTAGACGACGAGGCAG<br>GTCCCCTAGAAGAAGAACTCCCTCGCCTCGCAGACGAAGGT<br>CTCAATCGCCGCGTCGCAGAAGATCTCAATCTCGGGAATCTC<br>AATGTGACTACAAGGATGACGATGACAAGGATTACAAAGAC<br>GACGATGATAAGGACTATAAGGATGATGACGACAAATAG

Table S3. Performance and inference speed of the baseline methods

**Pearson R**	**Trail 1**	**Trail 2**	**Trail 3**	**Trail 4**	**Trail 5**
Our model	0.889353	0.883366	0.886623	0.891143	0.886344
MTtrans	0.889068	0.887611	0.89522	0.889258	0.886806
5' UTR LM	0.84037	0.85572	0.83659	0.8437	0.85042
Optimus 5-Prime	0.847104	0.83909	0.84609	0.851971	0.846592
FramePool	0.8128	0.81741	0.80574	0.80722	0.80903
	**Trail 6**	**Trail 7**	**Trail 8**	**Trail 9**	**Trail 10**
Our model	0.89191	0.885532	0.88424	0.890889	0.893929
MTtrans	0.887421	0.877455	0.886932	0.89135	0.884164
5' UTR LM	0.84689	0.83967	0.84578	0.84632	0.8373
Optimus 5-Prime	0.854622	0.845557	0.829584	0.845288	0.851266
FramePool	0.81308	0.80204	0.8034	0.81072	0.81407
**Spearman R**	**Trail 1**	**Trail 2**	**Trail 3**	**Trail 4**	**Trail 5**
Our model	0.873091	0.875925	0.878317	0.876237	0.877311
MTtrans	0.874583	0.877151	0.87995	0.87734	0.874692
5' UTR LM	0.83642	0.84271	0.82214	0.83523	0.83756
Optimus 5-Prime	0.829074	0.832198	0.840479	0.838285	0.838123
FramePool	0.79395	0.79701	0.78382	0.78941	0.79223
	**Trail 6**	**Trail 7**	**Trail 8**	**Trail 9**	**Trail 10**
Our model	0.881076	0.873038	0.869778	0.875901	0.878832
MTtrans	0.873272	0.87479	0.87214	0.880341	0.873142
5' UTR LM	0.82109	0.82154	0.83467	0.8316	0.82145
Optimus 5-Prime	0.846441	0.832853	0.817339	0.830282	0.838732
FramePool	0.79812	0.79195	0.78852	0.79426	0.79248
**Time Consume**	**Trail 1**	**Trail 2**	**Trail 3**	**Trail 4**	**Trail 5**
Our model	7.923	7.952	7.945	7.957	7.966
MTtrans	9.921	9.935	9.966	9.98	9.96
5' UTR LM	15.682	15.67	15.74	15.684	15.688
Optimus 5-Prime	5.747	5.742	5.748	5.774	5.692
FramePool	7.632	7.646	7.655	7.68	7.697
	**Trail 6**	**Trail 7**	**Trail 8**	**Trail 9**	**Trail 10**
Our model	7.927	7.941	7.956	7.944	7.938
MTtrans	9.973	9.937	9.933	9.943	9.979
5' UTR LM	15.673	15.684	15.697	15.645	15.624
Optimus 5-Prime	5.764	5.744	5.76	5.741	5.775
FramePool	7.641	7.714	7.66	7.634	7.635

Table S7. Statistical analysis for the altered nucleotides

**Altered Counts**		
**Base**	**Counts**	**Proportion**
Ta	8873	0.178301583
Ca	7953	0.159814324
Tg	4733	0.095108914
Ct	4637	0.093179809
Cg	4316	0.086729363
Gt	4264	0.085684431
Ga	3777	0.07589824
At	3765	0.075657102
Ag	3301	0.066333092
Gc	2461	0.04945342
Ac	1445	0.029037055
Tc	239	0.004802669
Total	49764	1
**Original proportion**		
**Base**	**Counts**	**Proportion**
A	8511	0.171027249
T	13845	0.278213166
C	16906	0.339723495
G	10502	0.21103609
Total	49764	1
**Optimized Proportion**		
**Base**	**Counts**	**Proportion**
A	20603	0.414014147
T	12666	0.254521341
C	4145	0.083293144
G	12350	0.248171369
Total	49764	1

Table S9. Primer and raw data for qPCR

Primer	Sequence						
h-β-actin-F	TAAGGAGAAGCTGTGCTACGTC						
h-β-actin-R	TTTCGTGGATGCCACAGGAC						
h-EGFP-F	CTACCCCGACCACATGAAGC						
h-EGFP-R	CTTGTAGTTGCCGTCGTCCT						
**Ct Data**								
**Sample**	**β-actin**				**EGFP**			
ori1-1	16.24	15.9	16.1	15.58	9.44	9.28	9.33	9.33
ori2-1	15.74	15.92	15.6	15.43	9.38	9.52	9.41	9.45
ori3-1	15.37	15.39	15.39	15.24	9.07	9.09	9.06	9.14
opt1-1	15.4	15.49	15.49	15.25	9.13	9.2	9.17	9.36
opt2-1	14.49	15.45	15.53	15.45	9.36	9.5	9.48	9.34
opt3-1	15.22	15.23	15.1	15.27	9.08	8.95	8.89	8.91
egfp-1	15.2	15.5	15.33	15.37	9.18	9.45	9.34	9.77
NC-1	15.74	15.95	15.5	15.57	24.92	25.61	25.26	25.32
ori1-2	15.57	15.7	15.69	16.01	9.21	9.3	9.24	9.15
ori2-2	15.24	15.59	16.04	15.47	9.18	9.44	9.44	9.35
ori3-2	15.54	15.73	15.29	15.34	9.22	9.08	9.09	9.21
opt1-2	15.54	15.62	15.53	15.59	9.08	9.17	9.09	9.3
opt2-2	15.76	15.58	15.95	15.63	9.38	9.49	9.46	9.4
opt3-2	15.28	15.49	15.91	15.26	9.05	9.1	9.14	9.18
egfp-2	15.58	15.41	15.46	15.35	9.42	9.6	9.46	9.49
NC-2	15.9	16.21	15.92	16.03	23.9	24.05	23.58	23.5
ori1-3	15.76	15.77	15.63	15.82	9.53	9.45	9.74	9.67
ori2-3	15.49	15.86	15.95	15.51	9.66	9.59	9.71	9.56
ori3-3	15.5	15.59	15.43	15.36	9.3	9.15	9.18	9.34
opt1-3	15.4	15.25	15.44	15.52	9.41	9.43	9.5	9.34
opt2-3	15.48	15.52	15.38	15.4	9.5	9.52	9.61	9.57
opt3-3	15.28	15.32	15.41	15.26	9.33	9.24	9.26	9.35
egfp-3	15.46	15.51	15.6	15.34	9.6	9.71	9.75	9.63
NC-3	16.09	16.15	16.04	16.08	22.19	22.14	22.14	22.15
ori1-4	15.52	15.48	15.4	15.3	9.07	9.16	9.12	9.19
ori2-4	15.44	15.52	15.46	15.28	9.26	9.31	9.33	9.3
ori3-4	15.55	15.49	15.27	15.23	9.05	9.01	9.02	9.08
opt1-4	15.4	15.39	15.36	15.43	9.14	9.15	9.2	9.08
opt2-4	15.42	15.67	15.53	15.54	9.3	9.32	9.24	9.05
opt3-4	15.66	15.39	15.41	14.93	9.07	9.13	9.01	8.99
egfp-4	15.76	15.93	15.83	15.83	9.7	9.61	9.55	9.57
NC-4	15.5	15.43	15.32	15.16	23.46	25.03	24.35	23.6
